# Local pharmaceutical research and development capacity in a developing country: a qualitative exploration of perspectives from key stakeholders in Ethiopia

**DOI:** 10.1186/s40545-022-00491-3

**Published:** 2022-11-24

**Authors:** Muluken Nigatu Selam, Samuel Abera, Helen Geremew, Eskinder Eshetu Ali

**Affiliations:** 1 Department of Pharmaceutics and Social Pharmacy, School of Pharmacy, College of Health Sciences, Addis Ababa University, Yared Street, College of Health Sciences Administration Building, Room No: 308, PO Box 9086, Addis Ababa, Ethiopia; 2 Droga Pharma PLC, Addis Ababa, Ethiopia

**Keywords:** Local manufacturing, Pharmaceutical industries, Drug development, African pharmaceutical sector

## Abstract

**Purpose:**

Despite its importance in ensuring sustainable healthcare, there are huge challenges with pharmaceutical research and development (R&D) especially for developing countries mainly due to the high investment costs naturally associated with such activities. In this regard, the pharmaceutical sector in Ethiopia, the most populous nation in East Africa, faces numerous challenges. The current study aimed at assessing the R&D capacity of the local pharmaceutical manufacturers from the perspectives of key informants working in the companies and supporting government offices and education institutions.

**Methods:**

A qualitative study design employing in-depth interviews using semi-structured interview guides with flexible probing techniques was used for data collection. The study involved purposively selected participants who represented major stakeholders such as managers in the R&D departments of pharmaceutical manufacturers, officers and leaders in concerned government agencies and researchers in a local university. All transcribed interviews were subjected to thematic analysis and the Qualitative Data Analysis software in family R (RQDA) was used for data analysis.

**Results:**

A total of 14 participants were involved in the study and three major themes were identified from the interviews. Current R&D capacity, opportunities and challenges for involvement in R&D were the major themes. Under current R&D capacity, the weak R&D status of local pharmaceutical plants and minimal university–industry linkage were identified. The challenges of pharmaceutical R&D in Ethiopia included weak governmental and managerial support; difficult procurement processes for R&D input; and the high cost of R&D. Availability of trainable human power and planned government incentives were identified as the opportunities.

**Conclusion:**

Overall, there is a low level of R&D capacity in local pharmaceutical industries and timely interventional strategies should be implemented through collaboration of academia, research institutions and pharmaceutical industries.

**Supplementary Information:**

The online version contains supplementary material available at 10.1186/s40545-022-00491-3.

## Introduction

As medicines are a very important, if not the most important, input in healthcare, the need for their uninterrupted supply is critical in ensuring universal access to healthcare [1]. Nearly 2 billion people have no access to essential medicines in the world and about 80% of these live in low-income countries [1, 2]. Ensuring sustainable access to medicines in developing countries requires supporting research and development (R&D) in addition to strengthening local capacities for manufacturing of medicines. That is why it is indicated as one of the main Sustainable Development Goals (SDGs) relating to Universal Health Coverage (UHC) [3]. In this regard, local pharmaceutical companies can play an important role to address the availability of essential medicines [2].

Nowadays, it is well-recognized that pharmaceutical companies play a critical role in enhancing health status worldwide. Successes for the pharmaceutical industries in expanding access to medicines are related to their research-based activities [4]. Pharmaceutical companies are constantly searching for new and improved drugs to treat diseases through R&D investment with the purpose of creating next-generation profit source or develop relatively cost-effective drugs [5, 6]. The bigger pharmaceutical companies ensured their continued existence through R&D and the main driver for the growth of the pharmaceutical industries is innovation [7, 8]. However, such companies are often criticized for not bringing more innovative medicines for the treatment of unmet medical needs especially for people in developing countries [5, 9]. The challenges for the pharmaceutical industries include declining internal R&D success rates coupled with patent expiries of important cash-generating drugs, governments pursuing austerity policies, and regulatory agencies tightening their demands. Due to such challenges of the R&D process, pharmaceutical companies, especially those in the developing parts of the world are dwelling much time on production of generic medicines giving less attention for the R&D activities for developing new drugs or improving the existing products [10, 11].

Despite Africa’s wealth of natural resources that can be used for the treatment and prevention of diseases, the burden of diseases is high in the continent. These natural resources provide an opportunity for African countries to develop new and/or improved drugs to curb a multitude of problems including neglected diseases for which most drug companies are reluctant to develop drugs. Despite this opportunity, there has been no significant development in drug discovery and development in many African countries. Thus, majority of these countries still rely on drugs developed by pharmaceutical firms in the developed countries [12]. Africa imports 70% of its pharmaceutical products [13] and the capacity for R&D and local drug production still has a long way to go. The biggest barrier to drug and vaccine technology transfer is lack of R&D capacity in developing countries. Moreover, lack of funds to invest in R&D, and failure by governments to create an enabling local environment of research infrastructure makes technology transfer less likely to succeed [14]. Furthermore, many African countries do not have the technical, financial or human resources required to engage in pharmaceutical R&D [13].

In Ethiopia, there are around 11 pharmaceutical industries engaged in large-scale manufacturing of generic medicines. The government has given due attention to the pharmaceutical sector in which establishment of the Kilinto pharmaceutical industrial park (KPIP) and the preparation of a 10 years national strategic plan of action (NSPA) for pharmaceutical manufacturing are among the measures taken so far for strengthening the sector. Creating an R&D platform is among the strategic objectives of the NSPA focusing on fostering high-quality research, facilitating technology transfer and promoting commercialization of R&D outputs into products and services [15].

The Ethiopian pharmaceutical companies also share similar challenges with companies in many other developing countries in terms of their R&D activities. The limited number of local companies together with their poor performance made the country heavily dependent on imported medicines for above 80% of its local demand. Given the indispensable role of R&D in ensuring self-reliance in pharmaceutical productions and supply, it is wise to explore the challenges of local pharmaceutical industries and supporting organizations towards R&D and identify solutions for the problems. The establishment and proper execution of R&D activities in the country requires a strong collaboration between the stakeholders in the state, research institutes and universities, and the private sector [15]. Hence, the current study aimed at assessing the status of the R&D units of the Ethiopian pharmaceutical industries and identifying the barriers and facilitators for the R&D activities by the companies.

## Materials and methods

### Study setting

This study was conducted through key informant interviews with experts and managers working in selected pharmaceutical industries located in Addis Ababa and its vicinity (a total of seven companies); relevant stakeholders from supporting organizations such as the Ethiopian Food Beverage and Pharmaceutical Industry Development Institute (FBPIDI), Ethiopian Investment Commission (EIC), Ministry of Innovation and Technology (MINT); the Ethiopian Pharmaceutical Manufacturers Association and research institutions such as Armauer Hansen Research Institute (AHRI) and Addis Ababa University (AAU). The study was conducted in April and May 2021.

### Study design

A qualitative study design employing in-depth interviews using semi-structured interview guides with flexible probing techniques was used.

### Sampling and participants

A purposive sampling strategy was used in this study. All of the participants had managerial positions and/or long experience in the area of conducting and or supporting R&D in pharmaceutical industries and research institutions. The key informant interviews were conducted with individuals with positions as R&D manager, plant manager, production manager, and quality assurance managers who are working in pharmaceutical industries. Moreover, key informants from the supporting stakeholders (MINT, EIC, FBPIDI), and an educational and research institutions like AAU and AHRI were also purposively sampled to take part in the study.

### Data collection

First permission was sought from the heads of the sampled organizations and consent was sought from potential participants. The most convenient date and time of interview was arranged in consultation with the consenting participant. One-to-one face-to-face in-depth interviews of key informants were conducted to collect the data from many of the participants. Some of the interviews were conducted through telephone calls. Before the actual interviews, objectives and process of the study were explained to the interviewees by the research team. The key informants read the participant information sheets (or it was read to them in case of telephone interviews) and were encouraged to raise questions about the study, which were answered accordingly. Participants gave verbal consent to take part in the study. A digital recorder was used to record interviews and the recordings were copied later to a computer and transcribed verbatim that facilitated coding and analysis.

One of the authors (MNS) conducted the interviews after receiving training on how to conduct qualitative interviews. Authors SA and HG pharmacists with experience in industrial pharmacy accompanied MNS during the interviews in order to facilitate note taking. Author EEA, an assistant professor of social and administrative pharmacy with training and experience in the conduct and analysis of qualitative studies supervised the interview process by listening to the recordings and giving suggestions for the next interviews. The in-person interviews took place in the participants’ offices.

Each interview lasted for approximately 20–35 min. The interviews were done in Amharic language using a semi-structured interview guide (Additional file 1) and to enrich the study objectives, probing questions were asked where appropriate to capture more detailed information on the issue involved. Demographic data of key informants were obtained before starting the interviews and this helped the interviewer to establish a better rapport with the respondent.

### Data analysis

Analysis of the interview data was done following the inductive thematic analysis approach outlined by Braun and Clarke [16]. The inductive approach was employed to gain in-depth understanding of participants’ opinions without having to subscribe to pre-defined theories or assumptions. Transcription of the audio-recorded interview in Amharic language was done. The transcripts were checked against the audio-records to ensure accuracy. Based on recommendations by Braun and Clarke [16], analysis in this study started with a repeated reading of the transcripts to ensure familiarization with the data and credibility of data sources. Generation of initial codes and analysis were made by the last author (EEA) based on interview transcripts. After the initial transcription and analysis were completed using the interview language (Amharic), the identified themes were then translated to the English language. As suggested by cross-cultural researchers, translations to Amharic were done later in the analysis in order to prevent lost meanings in translations [17]. Identified codes were then sorted into themes by using thematic maps to depict potential relationships between themes and subthemes. Then, the identified themes were reviewed and refined to ensure coherence between the themes, coded extracts and the entire dataset. Constant meetings of the investigators were held to ensure the accuracy of the interpretations by the coder and facilitate confirmability of the analysis. Moreover, member checking was done by emailing the final draft of the summarized themes and corresponding quotes to study participants. All the participants confirmed that the researchers’ interpretations were fair and appropriate representations of the views they reflected during the interviews. Detailed characteristics of participants are provided to facilitate transferability of the findings. The Qualitative Data Analysis software in family R (RQDA) was used in the process of data analysis [18].

## Results

### Participant characteristics

A total of 14 interviews were conducted. Twelve of the participants were male and six had a bachelor level education while the rest had a masters or PhD level education. Nine of the participants came from seven (63.6%) of the total of 11 pharmaceutical industries in Ethiopia. Eleven were pharmacists by profession. The majority (13) had over 10 years of overall working experience. In terms of experience at current position, 8 of the participants had less than 5 years of experience (Table 1).

**Table 1:** Participants’ characteristics

Characteristics	*n*
	14
Gender	
Male	12
Female	2
Age (years)	
30 – 35	3
36 – 40	3
41 – 45	3
46 – 50	4
51 – 55	1
Educational level	
Bachelor	6
Masters	6
PhD	2
Work setting	
Industry	9^a^
Government agency	3
Research/educational institute	2
Professional background	
Pharmacy	11
Chemical engineering	1
Biochemistry	1
Immunology	1
Work experience in current position (in years)	
< 5	8
5–10	5
11–15	1
Total years of work experience	
< 10	1
10–15	5
16–20	4
21–25	4

^a^This number includes 1 participant who is also a member of the leadership of the Ethiopian Pharmaceutical Manufacturers Association

### Findings of thematic analysis

Thematic analysis identified three main themes in the areas of: current R&D capacity, opportunities and challenges for involvement in R&D. Detailed descriptions of the findings under each theme are presented below.

### Current R&D capacity

This theme describes the situation in Ethiopia with respect to pharmaceutical R&D activities of the different stakeholders including pharmaceutical plants, research/educational institutions and government agencies. This theme is further classified into the subthemes: status in pharmaceutical plants and university–industry linkage.

### Status in pharmaceutical plants

According to participants, although there are good signs that some companies are heading towards a more R&D intensive practice, the situation in Ethiopia is that R&D is not given much attention by pharmaceutical manufacturers. When available, the R&D departments in the pharmaceutical plants in Ethiopia are limited to formulation development. The plants are not engaged in new drug development, excipient development, etc. The quotes in Table 2 further describe the details of what participants thought about the current practice.

**Table 2:** Subthemes on participants’ opinions regarding the status of R&D in Ethiopian pharmaceutical plants

Subtheme	Focus area	Quotes from participants
Status in pharmaceutical plants	Overall opinion about status	“From the currently active local manufacturers, may be two or three have a separate department for [R&D] formulation optimization, stability studies and similar activities” [Participant 06, from pharmaceutical manufacturer] “I would say that it is 0%… because there is no such research and development going on in Ethiopia… may be there is some formulation development done… that is within…the factory… but formulation development… whatever it is going on within the company… mostly the troubleshooting activity is going on rather than…. formulation development….” [Participant 11 from pharmaceutical manufacturer] “If you see each factory, they face product defect complaints during post marketing surveillance. But, they are not engaged in activities to study the reason for such defects. For example, if they want to change packaging… they do not have the capacity to search and find suitable material from local market, update their dossier application, get approval and continue production using the new material.” [Participant 01 from government agency]
	Types of pharmaceutical plants with respect to R&D capacity	“For us, R&D is the wing that greatly help us in cost minimization. As I told you in the beginning, we introduce new products through R&D only” [Participant 06 from pharmaceutical manufacturer] “We develop formulation in-house, by purchasing raw materials, developing the formula, doing the study, preparing the dossier, processing the registration. What we buy from foreign companies is the formulation for injectables… we currently have full dossier contract. But, such formula requires customization… because their machines and ours are different… so the formula needs to be customized for our facility and setup…” [Participant 12 from pharmaceutical manufacturer] “For all our products, the formulation, developmental study, all the data is taken from our parent company. So, we currently do not have R&D department.” [Participant 02 from pharmaceutical manufacturer]
	Concerns on external sourcing of formulation development	“Some companies are multinational… the main R&D activity is done by the mother company. Here… they only mix and the challenge is that there will be no technology transfer. Especially, there will be huge problem when a problem arises in the production process as the experts and technicians cannot solve the problem. In my experience, companies that have R&D units have low recall rate when compared to those that do not have in-house R&D” [Participant 05 from pharmaceutical manufacturer] “Most of the local medicine manufacturers do not take R&D seriously. Moreover, companies with joint ventures have their R&D units in the parent company. This has made their R&D activities dependent on the parent company. Generally, local medicine manufacturing companies have very weak R&D work” [Participant 03 from government agency]
University–industry linkage	University–industry linkage	“The goal of research institutions is publication. Until recently, what we used to count as an output is the number of publications that we have. I have supervised masters and PhD students but their research did not go any further after publication. We never had the habit of translating research findings to something useful…” [Participant 08 from research/educational institution] “I don't know how useful the research output of universities is for industries that largely engage in formulation activities. That is why I say the research is not practical. They [universities] also do research on novel drug delivery… targeted delivery… liposomes and the like… which is not applicable…” [Participant 06 from pharmaceutical manufacturer] “Industries do not understand the value of R&D… they consider doing their routine production activities as a major success. As a result… they think pursuing collaborations with universities would lead to financial or time loss… they think it does not have any value” [Participant 04 from government agency]

According to participants, there are various types of pharmaceutical plants. The first type is a company with no parent company outside Ethiopia. There are also companies, which are subsidiaries to pharmaceutical companies from China, India and the United Arab Emirates. Depending on the type of the company, their level of engagement in the formulation development activities varies. There are some which are self-reliant in terms of developing formulations for medicines, which are not in their product portfolio; there are others that buy the formula from external sources and customize it to their situation; there are also others that are fully reliant on their parent companies outside Ethiopia. In some cases, some local factories use both options of in-house formula development and buying of formula from outside sources. Refer to Table 2 for quotes that describe participants’ opinions in this regard.

Some participants raised concerns with the over-reliance of most of the pharmaceutical plants on the formulation development work done by external companies. They believe that this practice would deprive the plants of in-house capacity to resolve problems by themselves. Some participants even went as far as claiming that product recalls are rampant among those companies which lack in-house R&D capability as compared to those that have such departments (Table 2).

### University–industry linkage

Overall, participants assessed the status of university–industry linkage in the Ethiopian pharmaceutical sector as weak. Participants mentioned the focus of research/educational institutions on publications (instead of the extent to which the research solved real life problems), lack of alignment of research topics to the ones that the industry can apply and the fact that the industries do not see any immediate advantage to linkages with universities. The quotes in Table 2 show participants’ assessment of the status of university–industry linkage.

### Opportunities

This theme describes the conditions that can potentially facilitate and encourage involvement in pharmaceutical R&D activities. The main subthemes here are related to government incentives, availability of trainable human power and planned but not implemented incentives.

### Government incentives

According to participants, most of the incentives provided by the government for R&D are those that are provided for the routine production activities, which are related to allowing companies to import materials and chemicals duty free. Government support in granting of work permits for R&D experts who come from abroad and research grant packages were also mentioned as incentives to engage in R&D (Table 3).

**Table 3:** Participants’ opinion regarding the opportunities for R&D in pharmaceutical plants of Ethiopia

Subtheme	Quotes from participants
Government incentives	“Devices, raw materials and chemicals can be imported duty free. We also facilitate work permits for foreign experts who come to work in R&D activities of the local medicine manufacturing companies” [Participant 03 from government agency] “There are research packages, for example there are big grants that amount up to five million birr… what I remember is researchers in our sector (pharmaceutical) are major beneficiaries of such grants.” [Participant 04 from government agency]
Availability of trainable human power	“I don't think there will be problem with human power. Production and quality control department in all facilities hire many professionals. Professionals who come from those departments can easily fit in R&D… people who complete their graduate studies can also fit. They may need some induction training but they can fit for the current situation in our country as we mainly focus on conventional dosage forms” [Participant 06 from pharmaceutical manufacturer] “If all of a sudden… 20 factories start work, there is no human resource… I tell you the truth… if 20 companies enter the market at a time… the first complaint will be that there is no human resource… there can be trainable individuals… but… there is none that can start work on day one” [Participant 05 from pharmaceutical manufacturer]
Planned but not implemented	“This strategic plan puts creation of a country with R&D intensive pharmaceutical manufacturers at the end of the ten years. The plan was prepared by benchmarking many countries… it was a very good thing… now that it has been 5 or 6 years since its launch… when we see it… I think it was a little ambitious” [Participant 14 from research/educational institution] “… for example… it says [proposed law] ‘if a company rolls out a product after doing its own R&D… the tax holiday shall increase…’ but this law is not yet implemented… the same law also talks about cost sharing for the salaries of expatriate R&D specialists by way of income tax reductions… […] such schemes have already been endorsed by the council of ministers but have not been put in to law by the parliament…” [Participant 01 from government agency]

### Availability of trainable human power

According to participants, the available manpower is enough for the current demand. While all agreed that it would be difficult to find enough R&D staff in case more pharmaceutical manufacturers join the market, the currently available number of professionals was deemed sufficient. The presence of easily trainable professionals and graduate programs in various aspects of pharmaceutical production was also considered an advantage (Table 3).

### Planned but not implemented

According to participants, the government introduces different strategies and incentive packages, but most are not implemented as planned. The 10-year national strategy and plan of action for pharmaceutical manufacturing development in Ethiopia which envisions the presence of research-intensive manufacturers by 2025; the “second schedule package” that is designed to help pharmaceutical manufacturers to benefit from tax holidays in return for investment on R&D are the two major encouraging moves by the government which failed to materialize. The quotes in Table 3 explain those ideas.

### Challenges for involvement in R&D

This theme reflects the major challenges that pharmaceutical companies and research institutions face when trying to involve in R&D activities for pharmaceutical product development. These challenges range from those that are not specific to R&D to those that are unique to R&D activities. Each aspect of the challenges is discussed under the following subthemes: challenges not specific to R&D, cost of R&D and absence of resource pooling, difficult procurement processes for R&D input, and weak governmental and managerial support.

### Challenges not specific to R&D

Participants from pharmaceutical industries claimed that the government considers R&D as the other aspects of production. According to participants, foreign exchange shortage is a major issue in the Ethiopian economy in general. Despite the government’s special treatment of the pharmaceutical sector, it is still difficult to get the necessary foreign exchange on time to acquire materials and supplies. As there is no special emphasis for R&D activities, any foreign exchange request for such activities takes similar lengths of time to that for other production activities. An additional challenge for R&D activities is the fact that many of the factories are operating below production capacity. This, according to participants, has made the industries to worry about survival rather than thinking about the long-term R&D investments. The quotes in Table 4 depict this information.

**Table 4:** Participants’ opinion regarding the challenges related to R&D faced by pharmaceutical plants in Ethiopia

Subtheme	Quotes from participants
Challenges not specific to R&D	“The problem we had in the last 4… 5 years led us to be unable to produce the products in our portfolio… the obvious reason is foreign currency shortage… so… if you have 30 products may be 5 or 10 are ‘hot cake’ products… so… investing the available forex on those products will be more feasible… due to that, I don't know how many people are ready to want to develop formulations through R&D” [Participant 06 from pharmaceutical manufacturer] “… As our productivity is very low… we focus on increasing productivity. You need money for R&D… you have to hire staff… arrange for facilities and equipment… so… I think that is the challenge… because we are struggling to maintain our production capacity, it is difficult to go to R&D” [Participant 10 from pharmaceutical manufacturer] “…if you are running on 70… 80% of capacity no problem… you can think about the future…. but if you are running on around 15% capacity… then… naturally… it will be a little difficult for the organization…” [Participant 10 from pharmaceutical manufacturer]
Cost of R&D and absence of resource pooling	“I told you the cost of R&D is high… my company engages in R&D because we think of the long term…. Now we have around 100 products from 20 originally… […]… it takes time… very much… to get one [product] out you need two years for development studies and registration… and they [the industries] don't want it… because they are business companies” [Participant 12 from pharmaceutical manufacturer] “…rather than in-house formulation development… we are at a point where it is easier to buy from abroad… in-house development is costly… so the situation pushes you to buy even the formulations from abroad…” [Participant 07 from pharmaceutical manufacturers] “…it is sad… what is done somewhere is duplicated… the duplication… snatching of human resources … if one good expert… a foreign trained expert is in Gondar… and another one is in Jimma… another in Wollo… they cannot do much… what I suggest is to bring them to one institute… that is how they can be stimulated to do research…” [Participant 08 from research/educational institute] “we know our universities… a masters or PhD student… to do research… he will have to go… can he really work at the university… you know… he spends his time going from one institute to another to find equipment…” [Participant 05 from pharmaceutical manufacturer]
Difficult procurement processes for R&D input	“… our big challenge is… first… because we buy input materials in small quantities… it is difficult to get them… for example if the amount you need for development is 10 kg… what you find in the market is products packed in 50 kg…100 kg weight… one product may require 10 excipients and you will face the same problem for all… in addition the suppliers do not value small quantity purchases… and some even try to put it as a precondition for working with them in the future” [Participant 12 from pharmaceutical manufacturer] “I have also received funding… I also know others have too… but the procurement mechanism to avail input necessary for research is a problem… especially for those that should be purchased from abroad… it is a big obstacle…” [Participant 08 from research/education institute]
Weak governmental and managerial support	“Top-level managers consider R&D as a luxury… R&D is a big source of growth… seeing the global experience…. Multinationals invest a lot on R&D… so if you think of growth… change…. R&D is vital… I think there is a gap in knowledge… most companies do not give attention to R&D…” [Participant 05 from pharmaceutical manufacturer] “…there is no support from the government… NGOs…to help professionals get better exposure on R&D… and the companies are not interested to fund such activities…” [Participant 12 from pharmaceutical manufacturer] “the concerned institute to strengthen R&D in pharmaceutical manufacturing companies does not provide enough support for example in the areas of human resource capacity building… this is because the institute itself did not build its capacity… quite to the contrary… its employees sometimes learn R&D from the pharmaceutical companies…” [Participant 03 from government agency]

### Cost of R&D and absence of resource pooling

Participants mentioned that there is high cost related to R&D activities in terms of acquiring raw materials and human resources. In addition to that the nature of the R&D poses its own challenges as well. For example, the amount of chemicals that are required in the development process is very small but it is difficult to purchase such products in small pack sizes from the international market. As a result, there have been cases where larger package sizes have been procured leading to wastage of the remaining quantity. Participants also complained about the fragmented nature of the research practice in the country and the absence of a mechanism to pool available resources in terms of experts, equipment and chemicals. The major issues raised were related to the fact that similar researches are done in various universities causing duplication of efforts, that it is difficult to find research equipment and chemicals at one place and the fragmented information sources. The quotes in Table 4 depict these points.

### Difficult procurement processes for R&D input

Participants described that procuring materials used as input for R&D—especially from the international market—was very difficult. This was described from two directions. The first one is related to the small quantity/volume of chemicals needed in the formulation development process. This challenge was related to the lack of attention by international suppliers to small volume purchase orders and the absence of small volume packaging for the chemicals. The second one was related to the complex government procurement procedures in case of public research institutions. See the quotes in Table 4.

### Weak governmental and managerial support

This is mainly related to participants’ opinions about the low government support for some activities and the minimal support for R&D by top-level management of pharmaceutical companies. The quotes in Table 4 explain these ideas.

## Discussion

The current study is one of the first attempts to illuminate the situation of local pharmaceutical R&D activities in the context of a sub-Saharan African country, Ethiopia. It provided in-depth information about the current status, opportunities and challenges of the Ethiopian pharmaceutical industries in terms of R&D activities. It is believed that the findings of the study can be used to influence policy-making in Ethiopia and similar African countries. This is especially important as many African countries lack pharmaceutical R&D capabilities and are largely reliant on foreign purchases for their pharmaceutical needs [12–14].

The findings of the current study suggest that there is a very weak R&D capacity in the Ethiopian pharmaceutical industries, despite the government’s efforts to support it. While the weakness in R&D can be taken as a reflection of the country’s overall level of economic development, the pharmaceutical industries and the government could have done better given the immense potential of the pharmaceutical market in Ethiopia. The reasons for the weak R&D capacity are multifaceted ranging from lack of commitment by top-level industry managers to more complicated issues of economical and technical capability. Some participants believed that companies with weak R&D capabilities face the problem of product recalls more often than those with relatively stronger R&D units stressing that it is in the best interest of the companies to engage in R&D activities. While correlating weak R&D capability with product quality could be considered rational, more structured investigations of the issue should be done to generate empirical evidence.

The local pharmaceutical industries are mainly focused on formulation development and trying to make minor improvements on existing products. Due to the long-term nature of research investments on new drug developments, there is inclination of pharmaceutical companies R&D activities on modified drugs and generic medicines as indicated by Lee and Choi [5]. The local pharmaceutical R&D problems are also further complicated by the weak university–industry linkage and over-reliance on foreign partners for formulation research. According to our participants, universities are perceived to engage in non-translatable and theoretical research. Apparently, there is weak research infrastructure in Ethiopian universities, which are themselves dependent on the pharmaceutical industry for critical equipment and research inputs. This remains to be the case despite the government’s attempts to introduce problem-solving research approaches in the public universities [19]. According to government strategy documents, R&D on pharmaceutical products is a major priority [15]. However, there is no clear data on annual government spending on pharmaceutical R&D in Ethiopia.

The findings also suggest that joint ventures with companies from outside Ethiopia have gaps in achieving the target of technology transfer envisioned by the plan of action for pharmaceutical manufacturing development in the country [15]. The relevant stakeholders should give attention to building the in-house capacities of such companies in a way that ensures their product formulation and development capabilities. Locally led-health research is very important to address the gap observed in providing solution to diseases that affect the poor. As such, joint ventures should try to address the issue of technology transfer to ensure sustainable pharmaceutical sector.

While there are numerous incentive packages that have been designed by the government, the participants of the current study indicated that many of such incentives have not been enforced. According to the participants, the delays in the enforcement of policies such as the “second schedule package” had their impacts on the slow progress of Ethiopian pharmaceutical companies’ towards becoming research intensive. In light of the ambitious targets set by the country’s plan of action for pharmaceutical manufacturing development, a lot still needs to be done in the area of strengthening R&D capabilities of local pharmaceutical industries [15].

The Ethiopian economy is generally known for foreign exchange shortages and its adverse impacts on livelihoods and private companies. The problem is even worsened by the current COVID-19 pandemic and the conflict in different parts of the country [20]. In this regard, the pharmaceutical sector is not an exception. According to our participants the foreign exchange shortage has forced the companies to perform well below 50% of their production capacity and in some cases this figure goes as low as 15%. Due to the acute shortage of pharmaceutical products in the country, the national pharmaceutical supply agency usually needs to make fast purchases. This creates a situation where the limited foreign exchange is allocated to imports of finished pharmaceutical products rather than the raw materials that are used to make them. In addition to the limited access to foreign currency, constraints related to technology and skilled manpower are among the reasons for operating below capacity. This will have various short-term and long-term implications with regard to profitability of the industries and investors’ appetite in the pharmaceutical sector of the country. Moreover, the impacts of the foreign exchange shortages will also be reflected in the R&D performance of the companies. Naturally, a company struggling for its survival may not be interested in long-term investments that require R&D activities. Furthermore, local producers compete with foreign manufacturers for the market and they must be able to sell at a lower price and remain profitable and viable. The current situation does not favor the local producers who have to bear the higher costs of technologies and raw materials to stay in compliance with good manufacturing practices and other regulatory requirements.

### Limitations

The current study involved major key informants and provided an in-depth analysis of the R&D situation in the Ethiopian pharmaceutical industry and can showcase the situation in other setting within sub-Saharan Africa. However, it is not without limitations. The fact that it is mainly a qualitative study meant that quantitative impacts of weak R&D capacity have not been enumerated. The reliance on respondents’ subjective opinions potential bias in the researchers’ interpretation of respondents’ opinions may limit the applicability of the findings. There is a further need for studies in other settings to ensure repeatability of the findings of the current study. Moreover, the study focused mainly on actors in the pharmaceutical sector. As such the perspectives of stakeholders such as those in the national bank and other aspects of the pharmaceutical supply chain have not been addressed in the study. Future studies are recommended to address those issues.

## Conclusion

The findings of the current study suggest that there is a very weak R&D capacity in the local pharmaceutical industry. Weak university–industry linkage, over-reliance on foreign partners for formulation research and the resulting absence of technology transfer and local capacity building; delayed/weak enforcement of government policies and the overall weakened profitability of the industries due to lowered production capacity as a result of foreign exchange shortages were the major reasons for the weak R&D practice in local pharmaceutical companies.

## Supplementary Information

**Additional file 1: **Data collection tool.

## Data Availability

All data generated or analyzed during this study are included in the manuscript and Additional file.

## References

[1] WHO. Access to medicines: Making market forces serve the poor. 2017. http://www.who.int/publications/10-year-review/en/. Accessed 15 Dec 2021.

[2] Ozawa S, Shankar R, Leopold C, Orubu S (2019). Access to medicines through health systems in low- and middle-income countries. Health Policy Plan.

[3] WHO. World health statistics 2019: monitoring health for the SDGs, sustainable development goals. 2019. https://www.who.int/publications/i/item/world-health-statistics-2019-monitoring-health-for-the-sdgs-sustainable-development-goals. Accessed 15 Dec 2021.

[4] Lenssen G, Arenas D, Lacy P, Pickard S, Sturchio JL (2008). Business engagement in public programs: the pharmaceutical industry’s contribution to public health and the millennium development goals. Corp Gov.

[5] Lee M, Choi M (2015). The determinants of research and development investment in the pharmaceutical industry: focus on financial structures. Osong Public Health Res Perspect.

[6] Alt S, Helmstädter A (2018). Market entry, power, pharmacokinetics: what makes a successful drug innovation?. Drug Discov Today.

[7] Grevsen JV, Kirkegaard H, Kruse E, Kruse PR (2016). Early achievements of the Danish pharmaceutical industry-8. Lundbeck Theriaca.

[8] Liu C, Constantinides PP, Li Y (2014). Research and development in drug innovation: reflections from the 2013 bioeconomy conference in China, lessons learned and future perspectives. Acta Pharm Sin B.

[9] Basavaraj S, Betageri GV (2014). Can formulation and drug delivery reduce attrition during drug discovery and development—review of feasibility, benefits and challenges. Acta Pharm Sin B.

[10] Forster SP, Stegmaier J, Spycher R (2014). Virtual pharmaceutical companies: collaborating flexibly in pharmaceutical development. Drug Discov Today.

[11] Congressional Research Service. Pharmaceutical Research and Development: A Description and Analysis of the Process. Institution; 2001. https://www.everycrsreport.com/reports/RL30913.html. Accessed 05 Feb 2021.

[12] Nyigo VA, Malebo HM (2005). Drug discovery and developments in developing countries: bottlenecks and way forward. Tanzan Health Res Bull.

[13] Pheage T. Dying from lack of medicines: Encouraging local production, right policies the way out. 2017. https://www.un.org/africarenewal/magazine/december-2016-march-2017/dying-lack-medicines. Accessed 11 Dec 2021.

[14] WHO. Local Production for Access to Medical Products: Developing a Framework to Improve Public Health. 2011. https://www.who.int/phi/publications/Local_Production_Policy_Framework.pdf. Accessed 10 Dec 2021.

[15] Ministry of Health and Ministry of Industry. National strategy and plan of action for pharmaceutical manufacturing development in Ethiopia (2015–2025): Developing the pharmaceutical industry and improving access. 2015. https://www.who.int/phi/publications/Ethiopia_strategy_local_poduction.pdf?ua=1. Accessed 10 Dec 2021.

[16] Braun V, Clarke V (2006). Using thematic analysis in psychology. Qual Res Psychol.

[17] Temple B, Young A (2004). Qualitative research and translation dilemmas. Qual Res.

[18] Huang R. RQDA: R-based qualitative data analysis. R Package Version 3.3.2. 2016. http://rqda.r-forge.r-project.org/. Accessed 07 Feb 2017.

[19] Woldegiyorgis AA (2017). The vicious circle of quality in Ethiopian higher education. Int Higher Educ.

[20] Alam M, Yigzaw GS. Causes and impacts of foreign currency reserve crises in Ethiopia Üniversitepark Bülten. 2020;9:15–27.

