# Supplementary material for: Local pharmaceutical research and development capacity in a developing country: a qualitative exploration of perspectives from key stakeholders in Ethiopia
Source: J Pharm Policy Pract. 2022 Nov 24;15:92. doi: 10.1186/s40545-022-00491-3 (PMC9700997; doi:10.1186/s40545-022-00491-3)
Supplement: Supplementary file 1 — Additional file 1: Data collection tool. [file 40545_2022_491_MOESM1_ESM.docx]

# Data collection tool

**Addis Ababa University**

**College of Health Sciences**

**School of Pharmacy**

**Department of Pharmaceutics and Social Pharmacy**

**Participant Information (Introduction)**

This questionnaire is prepared for in-depth interview to identify the perception of pharmacy professionals in industries and policy makers towards Research and Development (R&D) activities for medicines. The research is also aimed at investigating the opportunities and barriers to local pharmaceutical plants in carrying out R&D activities. This study is conducted by AAU, College of Health Sciences, School of Pharmacy, Department of Pharmaceutics and Social Pharmacy.

Your participation is purely voluntary and information you provide will be kept completely confidential. Your name will never be written and aggregate responses from different respondents are identified only by codes. However, tape recorder may be used during the interview.

Your honest response to the questions is of paramount importance for the successful completion of the study. There is no Right or Wrong answer and you can have clarification for any doubt regarding the questions.

Are you willing to respond to the questions? Yes 🞏 No 🞏

**If you say yes, the Interview begins**

Date of interview: _____________________

**Part one: Background Information on Participants**

1. Age: ____________
2. Gender Male Female
3. Educational background

College diploma

B.Pharm/B.Sc

M.Pharm/M.Sc

PhD

1. Profession (Pharmacist, Chemist, etc) ____ ____________
2. Practice setting (Industry, MINT, FBPIDI, EFDA, MOH, EIC, EMA, etc) __________
3. Present position of the interviewee: __________________
4. Years of total working experience: ____________
5. Years of working experience in present position: ___________
6. Field of specialization (if any): ______________________________

**Part two: Themes for discussion**

**A. For participants working in the local pharmaceutical industries**

1. How do you describe the overall R&D situation in pharmaceutical industries in Ethiopia?
2. How do you assess the overall R&D activities in your company?

Probe: Please tell me a little bit more about whether your company has an independent department for R&D [if not, why not and any plan in the near future], the staff distribution, budgeting for R&D, patents you own, etc

What are the main areas of activities in your company’s R&D unit? (New products or modifications of existing products, formulation development/optimization, Excipient development, biotechnology etc.)

How do you compare your company to others in Ethiopia and what are your reasons/basis for your assessment?

How do you compare your company to others in the rest of the world and what are your reasons/basis for your assessment?

1. In your opinion, what conducive conditions (incentive packages) are available for pharmaceutical industries in Ethiopia to engage in R&D activities?

Probe: availability of financial incentives, appropriate equipment, reagents, adequate staff, training related to R & D, university industry linkages, support from senior management and government.

What is your experience in making use of the incentives?

1. In your opinion, what are the barriers for pharmaceutical industries in Ethiopia to engage in R&D activities?

Probe: availability of financial incentives, appropriate equipment, reagents, adequate staff, training related to R & D, university industry linkages, support from senior management and government.

What is your experience in facing such barriers and how did you tackle them?

1. In your opinion, what should be done by the relevant stakeholders (government agencies and pharma companies) to support R&D activities of local pharmaceutical industries?
2. From your point of view, are there any relevant aspects or questions that you feel should be addressed, but weren’t mentioned thus far?

**Thank you very much for your time and consideration**

**B. For participants working in government agencies (MINT, FBPIDI, EFDA, MOH, EIC, EMA)**

1. How do you describe the overall R&D situation in pharmaceutical industries in Ethiopia?

Probe: How do you compare Ethiopian companies to others in the rest of the world and what are your reasons/basis for your assessment?

1. How do you assess the overall activities of your ministry/agency/office to support R&D activities in local pharmaceutical companies?

Probe: Please tell me a little bit more about the overall organizational structure (whether your ministry/agency/office has an independent department for supporting R&D in local pharmaceutical companies [if not, why not and any plan in the near future], the staff distribution, budgeting for R&D, etc.

What are the main areas of activities in your ministry’s/agency’s/office’s unit that supports R&D in pharmaceutical companies? (training, financing, etc.)

1. In your opinion, what conducive conditions (incentive packages) are available for pharmaceutical industries in Ethiopia to engage in R&D activities?

Probe: availability of financial incentives, appropriate equipment, reagents, adequate staff, training related to R & D, university industry linkages, support from senior management and government.

Which ones are provided by your ministry/agency/office?

1. In your opinion, what are the barriers for pharmaceutical industries in Ethiopia to engage in R&D activities ?

Probe: availability of financial incentives, appropriate equipment, reagents, adequate staff, training related to R & D, university industry linkages, support from senior management and government.

Which ones are related to your ministry/agency/office?

1. In your opinion, what should be done by the relevant stakeholders (government agencies and pharma companies) to support R&D activities of local pharmaceutical industries?
2. From your point of view, are there any relevant aspects or questions that you feel should be addressed, but weren’t mentioned thus far??

**Thank you very much for your time and consideration**
